# Oral palisaded encapsulated neuroma; a diagnosis seldom suspected clinically

**DOI:** 10.1002/ccr3.8212

**Published:** 2023-11-16

**Authors:** Sara Pourshahidi, Pouyan Aminishakib, Negin Aliyari, Kimia HafeziMotlagh

**Affiliations:** ^1^ Department of Oral and Maxillofacial Medicine, School of Dentistry Tehran University of Medical Sciences; ^2^ Oral and Maxillofacial Pathology Department, School of Dentistry Tehran University of Medical Sciences Tehran Iran; ^3^ Cancer Institute Hospital, IKHC Tehran University of Medical Sciences Tehran Iran; ^4^ Department of Oral Medicine, School of Dentistry Tehran University of Medical Sciences Tehran Iran

**Keywords:** nerve sheath neoplasm, neuroma, soft tissue neoplasm

## Abstract

**Key Clinical Message:**

Palisaded encapsulated neuroma (PEN) is generally seen in the head and neck area as an asymptomatic nodule with the same color as the surrounding skin and rarely occurs in the oral cavity. The exact etiology of PEN is not known, but there is evidence supporting the role of trauma as its etiological factor.

**Abstract:**

Palisaded encapsulated neuroma (PEN) is one of the benign nerve sheath tumors of Schwann cell origin, which is commonly found in the skin of the head and neck area, and rarely occurs in the oral cavity. Its exact etiology is unknown, but there is evidence that supports the role of trauma as an etiological factor. Here we present a case of PEN in the hard palate of a 30‐year‐old patient and review the differential diagnoses of these nerve sheath tumors of the oral cavity.

## Graphical abstract

PEN is a benign tumor of neural origin, a diagnosis seldom suspected clinically.

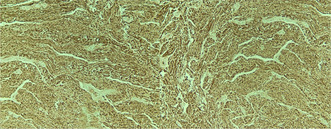

## 1 INTRODUCTION

Solitary circumscribed neuroma (SCN), which is also known as palisaded encapsulated neuroma (PEN), is a less known tumor with the origin of Schwann cells and axons, which mostly occurs in the skin.^1^ PEN is generally seen in the head and neck area as a single, asymptomatic nodule or papule with the same color as the surrounding skin. There is a tendency to involve the skin of the face (in more than 90% of the reported cases), and the nose and cheeks are known as the most common areas of PEN incidence.^2^ Rare cases of this lesion have also been reported in the oral cavity, eyelid, and penis.^3^, ^4^

This tumor is considered a type of small peripheral nerve fibers proliferation with or without a specific capsule, which can rarely have a plexiform or multinodular pattern.^5^ PEN's male and female ratio is the same. Although PEN can occur at any age, it is mainly found in middle‐aged adults.^1^ The exact etiology of PEN is not known, but there is evidence that supports the role of trauma as its etiological factor.^6^

There are also rare reports of multiple PEN occurrences, in which case genetic disorders such as phakomatosis are considered differential diagnoses.^7^ Since PEN has many similarities with schwannoma and neurofibroma, its diagnosis is challenging for clinicians. It is very important to distinguish PEN from neurofibroma because neurofibroma can be a part of neurofibromatosis 1 (Von Recklinghausen disease), which is a systemic disease with serious complications caused by neurofibroma of the peripheral and central nervous system.^8^

## 2 CASE PRESENTATION

**FIGURE 1:**
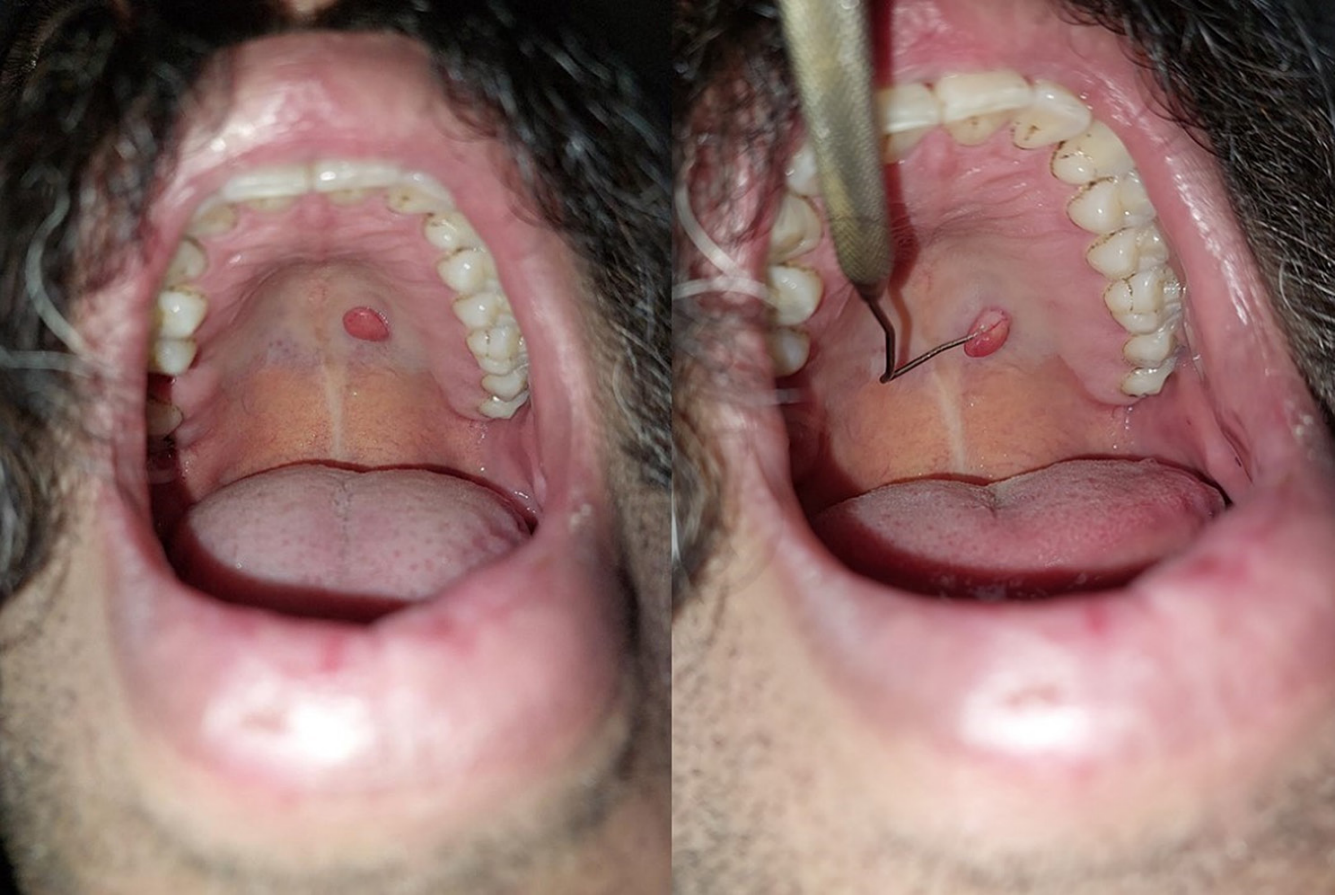
Intraoral view showing an exophytic pedunculated mass on the left side of the hard palate.

A 30‐year‐old male patient who complained of a mass in the palate referred to the Department of oral medicine. In the clinical examination, an exophytic mass with a diameter of about 1 cm with a smooth and telangiectatic surface was observed (Figure 1). This red‐pink mass was located on the left side of the hard palate and unilaterally to the midline. The lesion was asymptomatic with a firm consistency. There was no history of trauma to the area. The patient did not mention any history of parafunctional habits (such as daily chewing of any beetle nut) or smoking. There was no comorbid systemic disease or history of taking any medications.

With a differential diagnosis of irritation fibroma, the patient underwent an excisional biopsy with local anesthesia, and the sample was sent for histopathologic evaluation in formalin.

**FIGURE 2:**
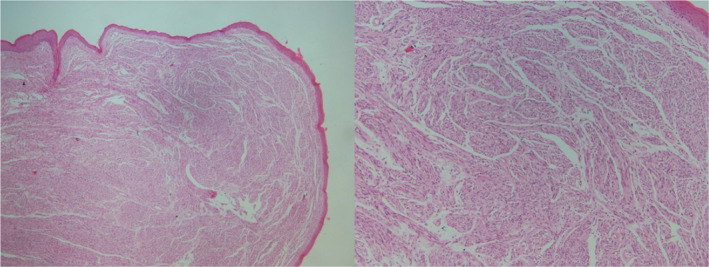
Microscopic sections showing well‐circumscribed, moderately cellular neoplastic tissue with a lobulated appearance. (H&E staining, ×40/×100).

**FIGURE 3:**
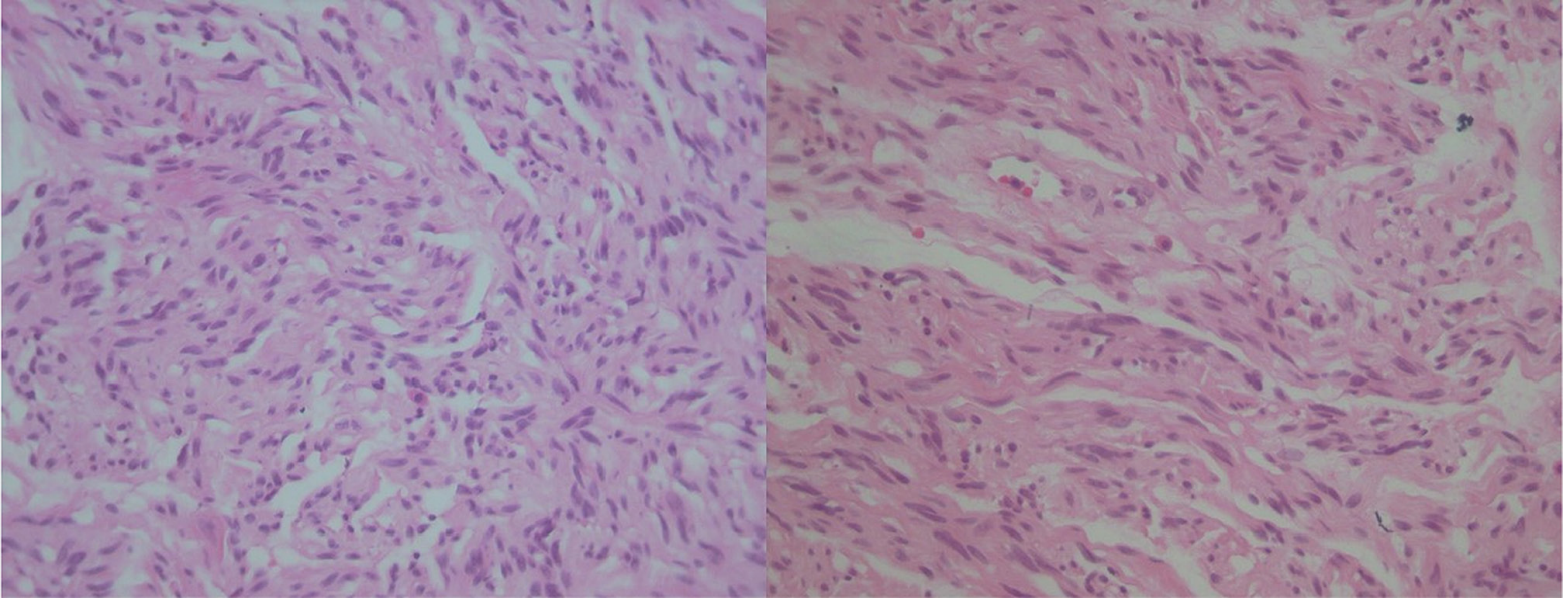
Microscopic sections showing bland‐looking spindle to wavy neoplastic cells arranged in interlacing fascicles (H&E staining, ×400).

**FIGURE 4:**
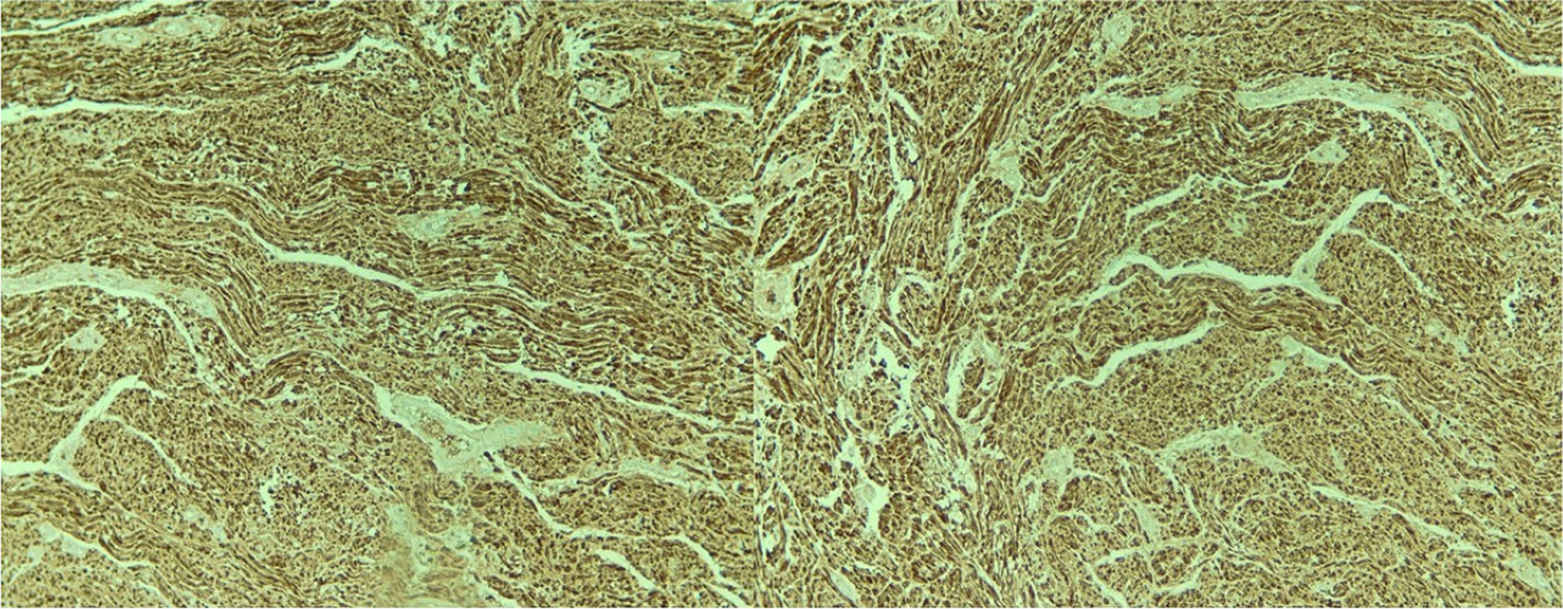
Neoplastic cells showing diffuse, strong immunoreactivity to S100 protein (×100).

In the histopathological examination, neoplastic tissue including well‐circumscribed sheets of wavy spindle cells of point‐end type was observed (Figures 2 and 3). No evidence of mitotic figures or nuclear pleomorphism is observed. The connective tissue was covered by atrophic epithelium without rete‐ridge and no evidence of cellular atypia or mitotic activity was reported (Figure 4). The cells showed a positive immunohistochemical reaction for the S‐100 protein. The final diagnosis was reported as bland‐looking spindle cell neoplasm, which matches with palisaded encapsulated neuroma.

The patient was called for follow‐up at 2, 4, and 8 weeks after surgery, and no evidence of lesion recurrence was observed in clinical examinations.

## 3 DISCUSSION

Palisade encapsulated neuroma (PEN) was first introduced as a benign neuroma in 1972 by Reed et al.^1^ Clinically, PEN appears as a solitary dome‐shaped, non‐pigmented nodule with a firm consistency on the face of adults. This tumor is known as a benign tumor of the facial skin and is rarely found in the oral cavity.^9^ PEN comprises only 0.04%–0.05% of oral biopsies. It is found in the oral cavity, often in the hard palate, gingiva, and labial mucosa of the maxilla, and occurs in middle‐aged adults in the fifth to seventh decades with equal prevalence in both sexes.^10^

Soft tissue oral cavity masses are often a diagnostic challenge because a diverse group of pathological processes can cause such lesions. Oral nerve tumors may be neoplastic or reactive in nature. These tumors consist of specific parts of peripheral nerves including endoneurium perineurium and epineurium.^11^

PEN is of the least common nerve tumors of the oral cavity and is often mistaken for schwannoma in microscopic view. Its histological variants include classic, lobular (the most common form), plexiform, and multilobular, which are clinically and biologically insignificant.^4^ As mentioned earlier, microscopic PEN is usually mistaken with schwannoma and less frequently with traumatic neuroma, as reported by Jordan et al. After reviewing the old slides, he classified 12 SCH and 4 traumatic neuromas as PEN.^12^

This lesion can be classified as classic or lobular, which is a more common histological variation, or plexiform, (fast growth rate like a mushroom), fungating, and multilobular. The cells are spindle‐shaped and arranged together in sheets, which indicates a palisading tendency. These sheets may bend immediately below the epithelium, together with the connective tissue. A specific finding is excessive artificial clefting as a result of tissue shrinkage. The stroma contains collagen and sometimes mucoid, and mast cells may be present in some lesions. Cells are positive for s100 and are always negative for GFAP. EMA in peritumor tissues non‐consistently stains not more than one cell layer. Some tumoral cells are also positive for CD34.^13^ The above‐mentioned characteristic findings were present in the case we reported. Neoplastic tissue including well‐circumscribed sheets of wavy spindle cells of point‐end type was observed and the cells also showed a positive immunohistochemical reaction for the S‐100 protein.

The most common oral nerve tumors are neurofibroma (NF), traumatic neuroma (TN), schwannoma (SCH) or neurilemoma, and palisaded encapsulated neuroma (PEN), which are included in the differential diagnosis of PEN, and differentiating these lesions is a diagnostic challenge.^14^

Neurofibroma is less common than schwannoma in the head and neck area, but the opposite is true inside the mouth. Neurofibroma is usually observed in the palate, gums, and tongue, and in the clinical view, is seen as isolated small sessile nodules or, with less prevalence, as multiple asymptomatic nodules that are covered with normal‐appearing mucosa.^15^ The risk of neurofibromatosis 1 (NF1) increases when multiple neurofibromas are seen in the oral cavity. In order to diagnose NF1, two of the following criteria must be present: 1. Six or more café au lait macules (more than 5 mm in diameter before puberty or less than 15 mm after puberty) 2. Two or more neurofibromas of any type or one plexiform neurofibroma, 3. A freckle in the armpit or groin, 4. Optic glioma, 5. Two or more lisch nodules (iris hamartoma), 6. A specific bone lesion such as sphenoid dysplasia or thinning of the cortex of long bones with or without pseudarthrosis, 7. Having a first‐degree relative with neurofibromatosis.^16^

In the histopathological view, nerve axons within the tumor stroma can be observed with silver staining. Cells are S100 positive in neurofibroma (Schwann cells), but not as much as in schwannoma. Some of them are also positive for epithelial membrane antigen (EMA) (perineurial cells), CD34 (endoneurial fibroblasts), Collagen IV (expressed by the basal membrane of nerve sheath cells), and CD68 (putative resident macrophages).^13^

The most common site of schwannoma is the head and neck region, but it is not so common in the oral cavity. Schwannoma is the third or fourth most common oral nerve tumor. It usually appears as a peripheral lesion, but sometimes it can occur inside the maxillary bone. The most common site of schwannomas manifestation includes the lips, especially the lower lip, as well as the buccal mucosa. Most cases are asymptomatic, but pain is reported in up to one‐third of cases. In rare cases, difficulty in swallowing, speaking, breathing, bleeding, macroglossia, or loss of sensation and taste may be reported by patients.^17^

Microscopically, conventional schwannomas are usually encapsulated and show cell proliferation in two different histological patterns and the most common one is Anthony A, which is characterized by spindle‐shaped cells surrounded by acellular eosinophilic areas, also known as Verocay bodies, and Antony B with cells and less organization, where the spindle cells are randomly placed in a loose myxomatous substrate.^18^

Traumatic neuroma (TN) is a reactive lesion and indicates a high growth response in response to nerve damage. Most TNs are peripheral, and only a small number of intraosseous traumatic neuromas have been reported in the English literature. TN usually manifests as an ulcerated nodule with a smooth surface on the tongue, mental foramen, and lip mucosa. Oral TNs are painful in 25%–30% of cases.^19^ In the histopathological view, this lesion is non‐encapsulated and is characterized by a number of axon bundles, nerve fibers, and Schwann cells in dense fibrous connective tissue. In IHC, the cells are s100 positive inside the nerve cells and more than 50% of them are positive for CD57 (myelin‐associated glycoprotein).^19^

## 4 CONCLUSION

Although neural neoplasms are uncommon in the oral cavity, these lesions should be considered in the differential diagnosis of oral masses, and a definitive diagnosis requires confirmation by histopathological and immunohistochemical examinations.

## AUTHOR CONTRIBUTIONS

**Sara Pourshahidi:** Conceptualization; project administration; supervision; writing – review and editing. **Pouyan Aminishakib:** Data curation; formal analysis; investigation. **Negin Aliyari:** Data curation; writing – original draft. **Kimia HafeziMotlagh:** Conceptualization; data curation; writing – original draft; writing – review and editing.

## FUNDING INFORMATION

The study was not funded.

## CONFLICT OF INTEREST STATEMENT

The authors declare that the research was conducted without any commercial or financial relationships construed as a potential conflict of interest.

## CONSENT

Written informed consent was obtained from the patient to publish this report in accordance with the journal's patient consent policy.

## Data Availability

The data that support the findings of this study are available on request from the corresponding author.
